# Microsurgical management of a brachial artery pseudoaneurysm in a 41-day-old infant

**DOI:** 10.1016/j.jvscit.2020.12.015

**Published:** 2021-01-28

**Authors:** Edgar Soto, Shivani Ananthasekar, Marc A. Passman, René P. Myers

**Affiliations:** a University of Alabama at Birmingham School of Medicine, Birmingham, Ala; b Division of Vascular Surgery and Endovascular Therapy, University of Alabama at Birmingham School of Medicine, Birmingham, Ala; c Division of Plastic Surgery, University of Alabama at Birmingham School of Medicine, Birmingham, Ala

**Keywords:** Anastomosis, Aneurysm, Brachial artery, False, Iatrogenic disease, Surgical

## Abstract

A pseudoaneurysm of the proximal right brachial artery is rare, with most caused by penetrating or blunt trauma. We report the case of a 41-day-old patient with a large iatrogenic pseudoaneurysm of the right brachial artery that had been induced by a puncture lesion during peripherally inserted central catheter placement for treatment of Lennox-Gastaut syndrome. The patient was successfully treated with a multidisciplinary approach, that consisted of direct excision of the pseudoaneurysm, followed by microvascular direct anastomosis. The patient was discharged with no complications, and complete exclusion of the pseudoaneurysm was confirmed at the 2-year follow-up examination.

Placement of a peripherally inserted central venous catheter (PICC) is often required in neonates to provide pharmacologic support.^1^ However, such insertion can lead to accidental puncture in the adjacent brachial artery, resulting in disruption of the arterial wall and blood dissecting into tissues, creating a perfused sac that communicates with the arterial lumen and risks pseudoaneurysm formation.^2^^,^^3^ Only 5% of arterial aneurysms in children will be found in the arteries of the upper limbs. The mainstay of treatment of upper extremity aneurysms remains prompt surgical resection owing to the risks of rupture and thromboembolic events distal to the lesions. We report an approach for treatment of a large false aneurysm of the proximal right brachial artery. The family of the patient provided written informed consent for the report of their child's case.

## Case report

**Fig 1:**
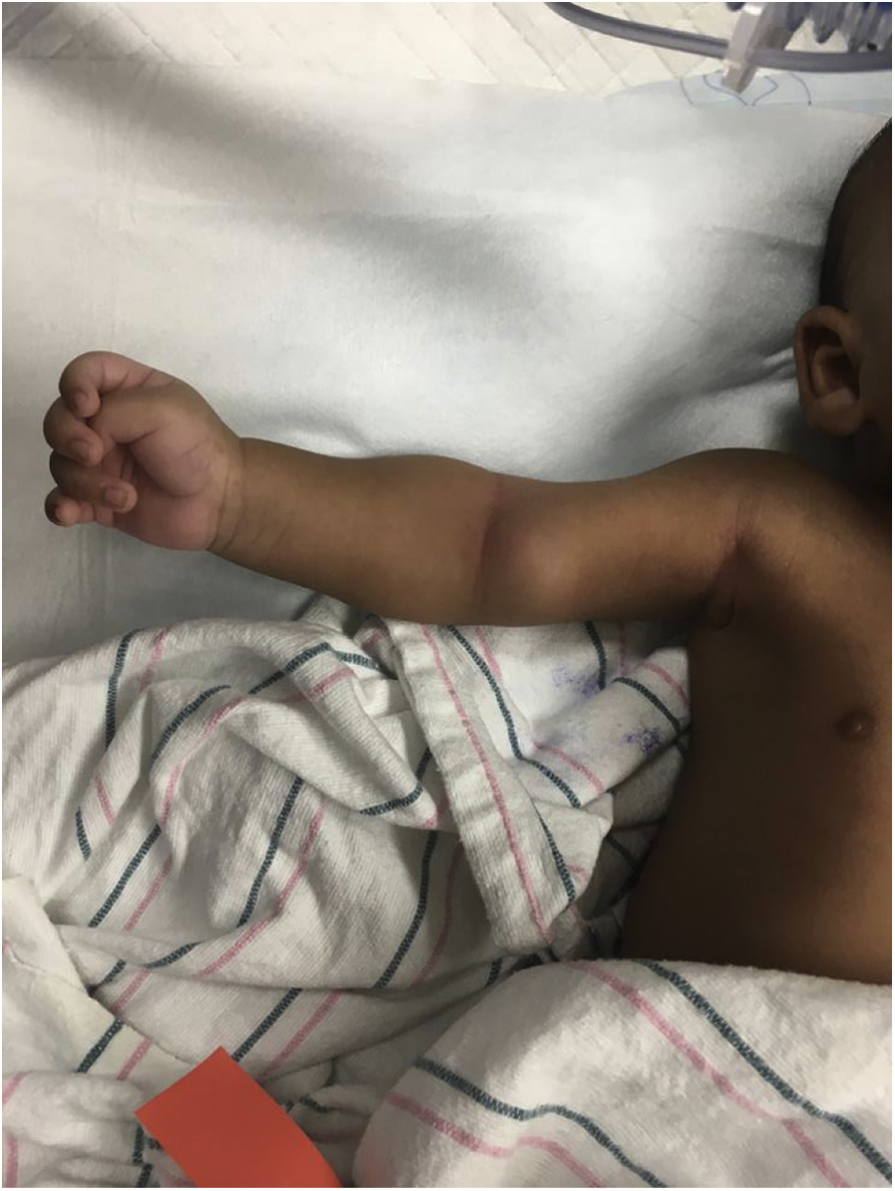
Photograph of a 41-day-old male infant showing the anteromedial pulsatile mass preoperatively.

A 39-week term, 41-day-old male infant with hypoxic-ischemic encephalopathy initially presented to the emergency department for evaluation of a rapidly enlarging mass located on the medial aspect of the right upper extremity. The mass was noted to be pulsatile with no effects on the radial and ulnar pulses. The patient had normal motor function, with full active movement of all extremities and mobility appropriate for his age. The patient's parents had noticed the mass 5 hours earlier and reported that it had been rapidly enlarging since then (Fig 1). The patient had undergone PICC placement in the affected area during the neonatal period for treatment of Lennox-Gastaut syndrome without status epilepticus.

**Fig 2:**
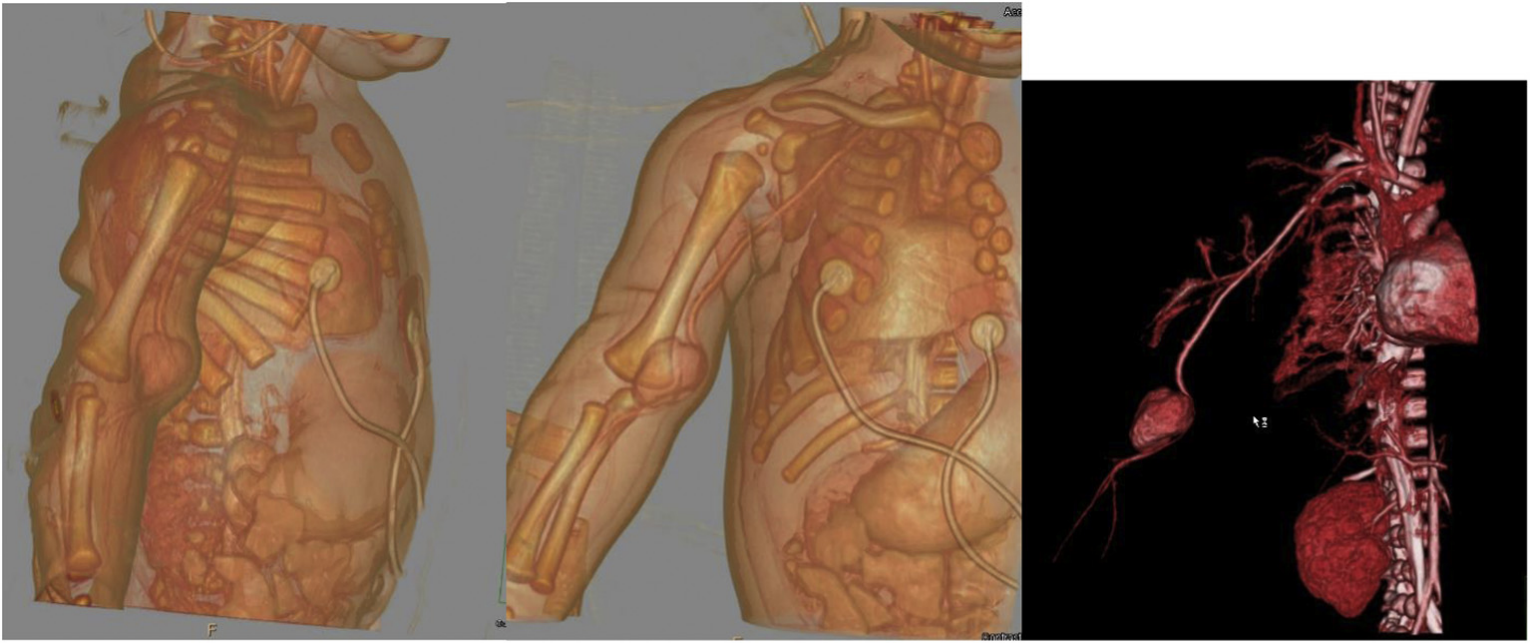
Three-dimensional computed tomography reconstruction of the right distal brachial artery pseudoaneurysm measuring 1.8 × 1.6 × 2.1 cm. The pseudoaneurysm communicated with the brachial artery and had contrast opacification of its lumen. No filling defect was seen within the pseudoaneurysm to suggest the presence of thrombus.

**Fig 3:**
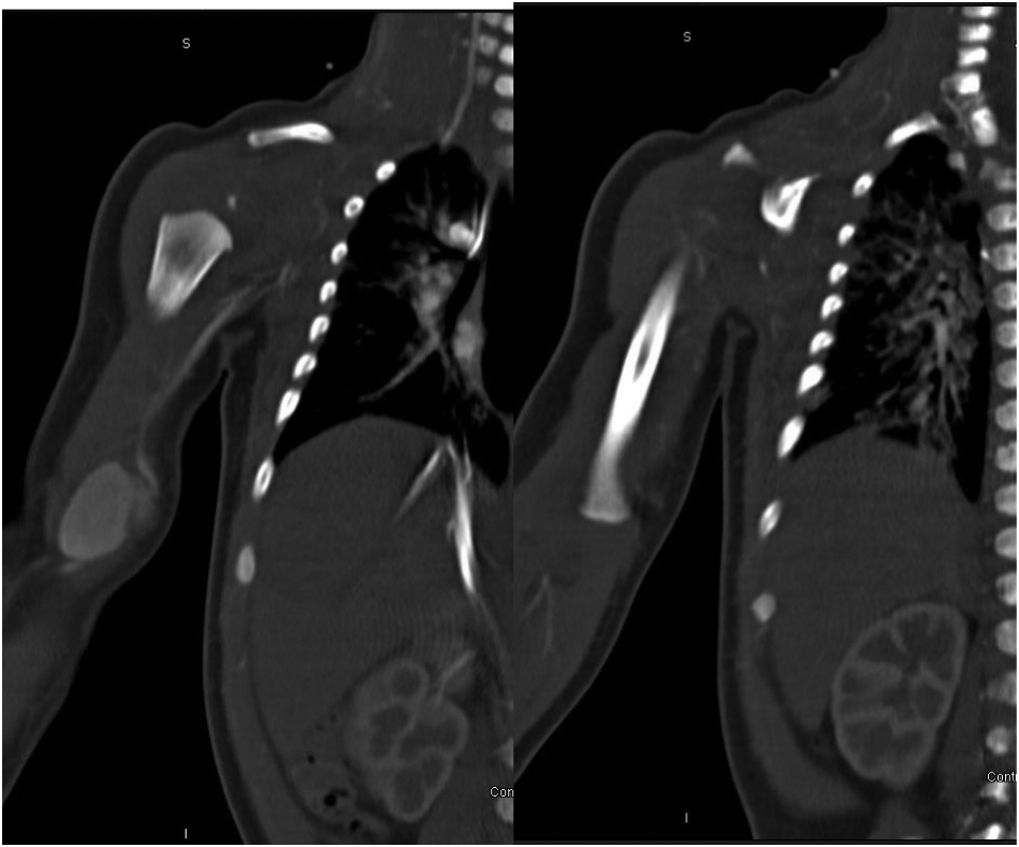
Contrast-enhanced magnetic resonance image showing a mass containing contrast at the medial aspect of the right brachial artery measuring 1.6 × 1.8 cm in cross section and 2.1 cm in craniocaudal dimension. Contrast opacification of the mass was present in the arterial phase secondary to communication with the brachial artery lumen. No solid contents were within the mass and no filling defects were present. The pseudoaneurysm extended to the subcutaneous soft tissue with no surrounding collection or edema seen.

The physical examination was notable for a pulsatile mass. Capillary refill occurred within <2 seconds. The pulses for all extremities were 2+, his extremities were warm, and he had no abnormal color and no edema. Computed tomography angiography showed a pseudoaneurysm of the right distal brachial artery measuring 1.8 × 1.6 × 2.1 cm (Fig 2). Communication between the pseudoaneurysm and the lumen of the brachial artery was seen, with no other vasculature affected and no other abnormalities. Further evaluation with contrast-enhanced magnetic resonance imaging showed a 2.1 × 1.7 × 1.1 cm mass along the right brachial artery with vascular flow (Fig 3). Because of concerns for a possible vascular abnormality from the previous iatrogenic injury, the patient was admitted to the neonatal intensive care unit for observation. During the next 2 days, the mass continued to grow, and the plastic surgery and vascular surgery divisions were both consulted.

Routine preparations for surgery were performed, and the parents provided written informed consent for microscope-assisted brachial artery repair planned by the primary plastic surgery team with intraoperative assistance from vascular surgery.

With the patient under general anesthesia, a curvilinear incision was performed over the ulnar aspect of the elbow. Next, the brachial artery pseudoaneurysm was exposed. Once the brachial sheath had been identified with dissection, the pseudoaneurysm was isolated from its superior and inferior attachments to the brachial artery. Once the pseudoaneurysm had been adequately mobilized, the brachial artery was dissected off the pseudoaneurysm down to the level of the defect. After clamping the proximal and distal vascular structures, the cavity of the pseudoaneurysm was entered and evacuated. The pseudoaneurysm pouch was completely resected and removed from its remaining attachment to the brachial artery. After the diseased segments of the brachial artery had been excised, the brachial artery was deemed conducive for direct microvascular end-to-end anastomosis to repair the brachial artery defect proximal to the antecubital fossa at which the vessel size was 2 mm. The repair was performed under an operating microscope with 9-0 nylon interrupted suture. No leak was noted when blood flow across the artery was restored. The artery was sprayed with papaverine, and a warm surgical sponge was placed over it for several seconds. This was a tensionless repair. Next, the patient underwent systemic heparinization. After hemostasis was achieved, the incision was closed. The blood loss was minimal at ∼15 mL. The surgery lasted 3 hours, 9 minutes.

**Fig 4:**
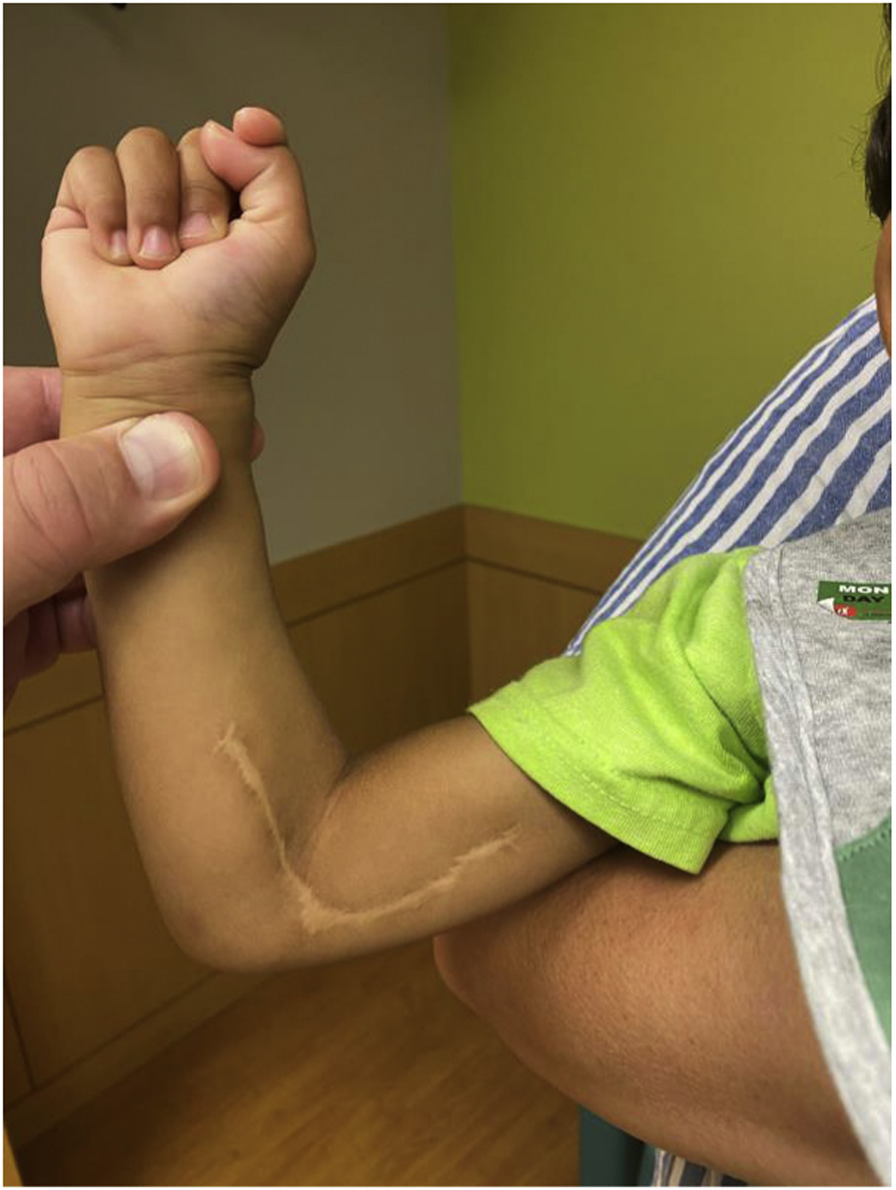
Photograph at 2 years postoperatively showing a well-healed scar.

In the postoperative period, the patient was given low-dose intravenous heparin (10 U/kg/h) for 48 hours. During the postoperative period, the patient was noted to have warm, dry, well-perfused, symmetric capillary refill in both upper extremities with no frank drainage or bleeding at the surgical site. After an uncomplicated postoperative course, the patient was discharged on postoperative day 3 with an arm splint and instructions to receive an oral suspension of acetaminophen 160 mg/5 mL. The patient was followed up by the plastic surgery service after 2 years, and the surgical site was well healed (Fig 4). The patient's hypoxic-ischemic encephalopathy led to complaints in the range of extension of the left upper extremity. However, the patient had better function with the right upper extremity, where the operation had occurred, than with the left upper extremity. The brachial, ulnar, and radial pulses were present bilaterally, and the wrist circumference was equal. The patient had a full range of motion in the right upper extremity without any neurologic deficits.

## Discussion

In children, upper limb artery injuries are extremely rare, with an incidence of false aneurysms of the brachial artery of ∼1% to 2%, and slightly more common than true aneurysms (0.13%).^2^ Iatrogenic injuries or penetrating injuries due to incorrect catheter insertion will cause breaks in the arterial lumen. Treatment is required to prevent amputation, rupture, and embolization.^4^^,^^5^

After delivery, our patient had presented with clinical signs of seizures. The patient received multiple antiepileptic drugs via the PICC. After stabilization, he was discharged and subsequently presented to the emergency department 40 days later with a pulsatile well-circumscribed mass. With the history of PICC placement, the false aneurysm was classified as an iatrogenic injury. The incidence of brachial artery pseudoaneurysms in this age group is extremely rare, with <20 recorded cases reported. This is especially true when no underlying congenital disorder or prematurity is present.^4^^,^^6^ Our patient had no signs of a vascular malformation or connective tissue disorder and no history of previous trauma.

We would recommend a multidisciplinary approach for the treatment of an iatrogenic pseudoaneurysm of the upper extremity in infants. In the present case, microsurgical repair was preferred because (1) aneurysmectomy could be performed before complications from thromboembolism could occur; and (2) surgical repair has been shown to rectify any impairment of limb growth resulting from a real or false arterial aneurysm in children.^7^ In addition, 9-0 nylon suture was used for the vascular anastomosis owing to the stability of its tensile strength and nominal tissue trauma. The interrupted suturing technique was chosen for its prevention of stenosis compared with running sutures.^8^ Ligation was not considered as a possible therapeutic approach for our patient because of the rapid limb growth at this age. Nevertheless, ligature might be indicated for an aneurysm distal to the profunda brachii artery or for a chronic thrombus that has completely occluded the vessel.^9^ Although it might be reasonable to observe small asymptomatic pseudoaneurysms, moderate or enlarging pseudoaneurysms have a greater risk of rupture, infection, and thrombosis leading to decreased circulation. Moderate, large, and enlarging pseudoaneurysms can be treated using microsurgical techniques to help prevent vascular and neurologic problems in infants and small children.

## Conclusion

To the best of our knowledge, our patient is the youngest patient with a large 1.8 × 1.6 × 2.1 cm brachial artery pseudoaneurysm reported. In pediatric patients, arterial aneurysms are rare and carry the risk of rupture and distal embolization complications. These patients can be adequately treated with a prompt diagnosis and microsurgical treatment.

## References

[1] Pet G.C., Eickhoff J.C., McNevin K.E., Do J., McAdams R.M. (2020). Risk factors for peripherally inserted central catheter complications in neonates. J Perinatol.

[2] Nurmeev I., Osipov D., Okoye B. (2020). Aneurysm of upper limb arteries in children: report of five cases. Case Rep Med.

[3] Mahady K., Thust S., Berkeley R., Stuart S., Barnacle A., Robertson F. (2015). Vascular anomalies of the head and neck in children. Quant Imaging Med Surg.

[4] Gow K.W., Mykytenko J., Patrick E.L., Dodson T.F. (2004). Brachial artery pseudoaneurysm in a 6-week-old infant. Am Surg.

[5] Žganjer M., Žganjer V., Cigit I., Čizmić A. (2012). Pseudoaneurysm of the brachial artery in a four-month-old boy: diagnosis and treatment. Arch Iran Med.

[6] Kesler W.W. (2019). Spontaneous radial artery pseudoaneurysm in an infant due to idiopathic medial hypoplasia—a case report. Case Reports Plast Surg Hand Surg.

[7] Pagès O.N., Alicchio F., Keren B. (2008). Management of brachial artery aneurisms in infants. Pediatr Surg Int.

[8] Calhoun T.R., Kitten C.M. (1986). Polypropylene suture—is it safe?. J Vasc Surg.

[9] Gangopadhyay N., Chong T., Chhabra A., Sammer D.M. (2016). Brachial artery aneurysm in a 7-month-old infant: case report and literature review. Plast Reconstr Surg Glob Open.

